# Leisure, Employment, Community Participation, and Quality of Life in Primary Caregivers of Autistic Children: A Qualitative Study

**DOI:** 10.1007/s10803-023-05992-x

**Published:** 2023-05-12

**Authors:** Gemma Davy, Josephine Barbaro, Katy Unwin, Cheryl Dissanayake

**Affiliations:** Olga Tennison Autism Research Centre, School of Psychology and Public Health, La Trobe University, Bundoora, VIC 3086 Australia

**Keywords:** Autism spectrum disorder, Caregivers, Occupational balance, Participation, Quality of life

## Abstract

**Purpose:**

In prioritising the needs of their autistic children, parents often modify their own participation across leisure, social, and workforce activities. Few studies have examined the impact these modifications have on caregiver quality of life (QoL). The aim in the current study was to examine how parenting their autistic child/ren impacts parent’s participation and QoL.

**Methods:**

Twenty primary caregivers (29–48 years, all female) of autistic children (7–11 years) were interviewed online about their participation in leisure, community, and employment activities including the impact of COVID-19.

**Results:**

Five themes with underlying subthemes were identified using reflexive thematic analysis. The themes were: (1) Reflecting on the important things in life, (2) Getting access to everything needed, (3) Barriers to participation in meaningful activities, (4) Facilitators of participation in meaningful activities, and (5) Participation through the COVID-19 pandemic.

**Conclusion:**

The findings highlight the importance of regular participation in meaningful activities for parents of autistic children and the support needed by them, particularly single parents, to achieve balance between meeting caring responsibilities and their own participation needs.

Parenting a child with additional needs requires responsibility beyond what is experienced by other families (Nelson, 2002). This can have a significant impact on the overall health and well-being of caregivers due to increased financial and time costs in providing care and making accommodations for their child (Miller et al., 2015; Stabile & Allin, 2012). The increased time pressure experienced by parents as they fulfill caregiving responsibilities and attend to their child’s needs has shown to impact negatively on caregiver quality of life (QoL; Bourke-Taylor et al., 2010), a multidimensional concept encompassing an individual’s overall health, well-being, and life satisfaction (WHOQOL Group, 1995). This outcome is particularly evident in caregivers of autistic children who commonly report increased parental distress and reduced QoL compared to parents of children with typical development or other developmental disabilities (Dabrowska & Pisula, 2010; Hayes & Watson, 2013).

Prior research examining parenting experiences in caregivers of autistic children highlights the increased time needed for caregiving related tasks (Safe et al., 2012), with parents reporting difficulty in combining care with daily activities (Chu et al., 2020; Ten Hoopen et al., 2020). Caregivers commonly report reduced autonomy, with daily tasks structured around their child’s needs and therapies (Joosten & Safe, 2014; Kim et al., 2018). When interviewed on their parenting experiences, mothers and fathers felt restricted in their ability to access desired activities and often needed to modify their participation levels to accommodate their child’s sensory or supervision needs (Bourke-Taylor et al., 2010; Dieleman et al., 2018; McAuliffe et al., 2019; Woodgate et al., 2008). These experiences are also reflected in time-use studies where mothers of autistic children report significantly more hours spent in caregiving tasks and fewer hours in leisure compared to mothers of neurotypical children (Smith et al., 2010). While the findings from these studies provide insight into the way families of autistic children allocate their time, the specific activities that parents identify as meaningful to their life, as well as the facilitators or barriers to accessing those activities alongside their caregiving responsibilities, are not clearly understood. Thus, further investigation is needed to identify potential areas of support that may assist families of autistic children in achieving balance between time spent caregiving and meeting their own individual needs.

Occupational balance refers to the extent a person’s daily activities and routines are fulfilling, purposeful, and in alignment with their values, priorities, and goals (Håkansson et al., 2006; Wagman et al., 2012). It involves finding a balance between the different occupations an individual may engage in (i.e., leisure, employment, self-care, and social activities); achieving that balance has been shown to promote overall health, well-being, and life satisfaction (Backman, 2004; Matuska & Christiansen, 2008). For parents of autistic children, achieving occupational balance may help to maintain a sense of fulfillment, satisfaction, and overall well-being by providing them with opportunities to engage in meaningful and enjoyable activities beyond their caregiving role (Davy et al., 2022). Further, it may also help them to better manage the time pressure associated with caregiving and to maintain their own physical and mental health. Therefore, examining caregiver participation with a particular focus on ability and/or desire to achieve occupational balance is important as the results from such investigation can strengthen our understanding of how to promote better outcomes for families of autistic children.

Although past research suggests parents tend to identify their ability to participate in their own interests and activities as an important predictor of their QoL (Bhopti et al., 2020), the beneficial impact of increased participation in various activities is not well understood. Improvements to parental QoL through increased participation in health-related activities has been demonstrated amongst mothers of children with additional needs, including autism (Bourke-Taylor et al., 2019; Niinomi et al., 2016). However, other research has identified increased frequency of leisure and social activities as being detrimental to maternal health related QoL (Rizk et al., 2011). Further, given the limited free time already reported by parents of autistic children, simply adding activities to their schedules may serve to increase the time pressure, burden, and fatigue experienced. Thus, an in-depth examination of caregiver participation and QoL needs, as well as the different factors that may influence their day-to-day lives, can help to provide clarity on what determines good QoL in parents of autistic children. It was for this reason a qualitative analysis was undertaken.

## Objectives

The aim in the current study was to examine caregiver participation in leisure, employment, and community related activities when raising autistic child/ren, and their perceptions and beliefs regarding their QoL. Primary caregivers were chosen as the focus for this study as they tend to take on the most responsibility when caring for an autistic child (McStay et al., 2014) and appear to be the most impacted in their day-to-day activities compared to secondary caregivers (Davy et al., 2022). Given reported differences in parenting school-aged autistic children or children with additional needs compared to typically developing children or autistic children from other age groups (i.e., preschool, adolescence; Callander & Lindsay 2018; McCann et al., 2012; McStay et al., 2014a), the current study focused on caregiver experiences in families of autistic children aged 7–11 years. The study was guided by the following research questions:

1. What do parents of school-aged autistic children consider as good quality of life?
2. How does parenting an autistic child impact caregivers’ ability to participate in a variety of leisure, employment, social, and community-based activities?
3. How does the parents’ participation experiences impact their quality of life?

## Method

### Study Design

To explore and capture the rich experiences and perspectives of primary caregivers of autistic children in navigating day-to-day life, an experiential qualitative research approach was adopted with individual semi-structured interviews conducted (Braun & Clarke, 2013; Madill & Gough, 2008). A critical realist framework was used to capture, analyse, and understand the participants lived experience (Braun & Clarke, 2013; Madill et al., 2000). The approach taken acknowledges the influence of social and cultural contexts on reality with an assumption that language is a simple reflection of articulated meanings and experiences (Braun & Clarke, 2013; Byrne, 2021).

### Participants

Participants were recruited from a longitudinal study examining parent and child outcomes in families of autistic children aged 7–11 years. Parents were recruited from unique cohorts of children, born between 2011 and 2013, who had decided to return for a follow-up assessment at school age. Children in Cohort 1 were diagnosed at 2-years following universal developmental monitoring in a community-based sample. See Barbaro et al., (2022) for a detailed description of the cohort. Children in Cohort 2 were also diagnosed at 2-years following clinical referral to an Early Assessment Clinic for Autism, which focused solely on children under 3-years. Children in Cohort 3 were diagnosed after 3-years and before 6-years of age as part of routine clinical referral and practise in the community. Twenty primary caregivers (all female) aged 29.90 to 48.62 years (*M* = 42.51, *SD* = 5.74) raising autistic children (80% male; aged 7.66 to 11.32 years; *M* = 9.36, *SD* = 1.32) were recruited (see Table 1). Sample size was informed by Braun and Clarke (2013) with saturation monitored by the first author to determine the end of data collection when no new meanings were interpreted (Guest et al., 2006; Morse, 1995, 2015).

**Table 1:** Demographic Characteristics of the Sample

	Parent Information				Autistic Child Information				
	Participant (age)	Marital Status	Education Level	Employment Status	Gender (age)	Schooling	AoD (months)	Additional Diagnoses	Siblings (ND)
P01	Mother (46)	Single	Diploma	Stay-at-home parent	Male (8)	Special developmental	29	ID, ADD	2
P02	Mother (43)	Married/de facto	Postgraduate	Casual	Male (11)	Mainstream private	17		1 (1)
P03	Mother (34)	Married/de facto	Diploma	Stay-at-home parent	Male (7)	Mainstream private	19	Language delay	2 (1)
P04	Mother (47)	Married/de facto	Diploma	Stay-at-home parent	Female (10)	Mainstream private	18	ADD	3 (3)
P05	Mother (45)	Divorced	Undergraduate	Part-time	Male (7)	Mainstream government	17	ID, AP, language delay	0
P06	Mother (33)	Married/de facto	Undergraduate	Part-time	Male (8)	Autism specific	26	Language delay	0
P07	Mother (41)	Separated	Diploma	Stay-at-home parent	Male (9)	Autism specific	20		0
P08	Mother (47)	Married/de facto	Undergraduate	Stay-at-home parent	Male (10)	Mainstream government	27		1 (1)
P09	Mother (48)	Married/de facto	Undergraduate	Part-time	Female (10)	Mainstream government	19	Language delay	0
P10	Mother (29)	Married/de facto	Undergraduate	Stay-at-home parent	Male (10)	Autism specific	33	ADHD	0
P11	Mother (42)	Married/de facto	Postgraduate	Full-time	Female (9)	Autism specific	25		1 (1)
P12	Mother (46)	Divorced	Postgraduate	Stay-at-home parent	Male (7)	Mainstream private	24	Language delay	2 (2)
P13	Mother (45)	Married/de facto	Postgraduate	Part-time	Male (10)	Mainstream government	29	ID, language delay	0
P14	Mother (35)	Married/de facto	Diploma	Stay-at-home parent	Male (9)	Mainstream government	18		2 (2)
P15	Mother (44)	Married/de facto	Undergraduate	Part-time	Male (9)	Dual enrolment	30		1 (1)
P16	Mother (48)	Divorced	Postgraduate	Full-time	Male (8)	ID specialist	20	ID, ADHD, language delay	0
P17	Mother (38)	Divorced	HS	Part-time	Male (7)	Autism specific	20		1 (1)
P18	Mother (47)	Married/de facto	Undergraduate	Casual	Female (10)	Mainstream private	28	ADD	2 (1)
P19	Mother (36)	Married/de facto	HS	Stay-at-home parent	Male (10)	Mainstream government	71	Language delay	1 (1)
P20	Mother (45)	Married/de facto	Less than HS	Stay-at-home parent	Male (7)	Mainstream government	23		1 (1)

*Note. ADD* = Attention Deficit Disorder, *ADHD* = Attention Deficit Hyperactivity Disorder, *AoD* = age of diagnosis, *AP* = Auditory Processing Disorder, *HS* = high school, *ID* = Intellectual Disability, *ND* = neurodivergent (e.g., ADHD, autism, dyspraxia

### Data Collection

Approval for this study was obtained from the Royal Children’s Hospital Melbourne and La Trobe University’s Human Research Ethics Committee (HREC/55792/RCHM-2019). After expressing interest in participating in an interview, parent participants reviewed the Participant Information Statement and provided informed consent.

Nine open-ended questions were developed to elicit information about caregiver experience, opportunity for participation in varying occupations (e.g., employment, leisure, social), QoL, and changes to participation in their own interests or activities that caregivers may have experienced throughout their child’s development (see Appendix for the Interview Questions). Data collection for the current study coincided with the peak of the COVID-19 pandemic in Victoria, Australia where stay-at-home orders were enforced, requiring schools and businesses to shut down for months at a time. To address this, questions were also included to capture the impact of the pandemic and state-wide lockdowns on caregiver participation and QoL. A semi-structured approach to the interview allowed participants opportunity to discuss issues that were important to them that may not have been anticipated by the researchers or captured by the interview schedule (Braun & Clarke, 2013).

In depth interviews were conducted with each participant by the first author via Zoom videoconferencing software. Due to the nature of the semi-structured approach, the duration of interviews varied due to the caregivers’ availability and their willingness to openly discuss their experiences, ideas, and beliefs. On average, the interviews conducted were 45 min in length. However, there were two interviews considered as outliers, with the shortest interview being 17 minutes in length and the longest at 94 minutes. Participants were provided with a cash voucher as compensation for their time.

### Data Analysis

Audio recordings of the interviews were transcribed initially through Otter.ai software (Otter.ai Inc, 2022) and then manually checked for accuracy by the first author. Each participant and their child/ren were allocated a pseudonym to maintain confidentiality. Following this, all participants received a copy of their transcript to clarify content and meaning, with no changes requested by participants.

Transcriptions were uploaded into NVivo 20 (QSR International Pty Ltd, 2020). Braun and Clarke (2006, 2013, 2022) reflexive thematic analysis was adopted allowing the data to be analysed in a manner that respects the subjectivity of participants’ experiences and attitudes while also embracing the researcher’s interpretations. An inductive approach to analysis allowed the themes and shared meanings between them to be grounded in the experiences of the participants rather than guided by past literature or the researcher’s own assumptions (Braun & Clarke, 2020).

Data analysis followed the six-phase process described by Braun and Clarke (2022). The first phase involved familiarisation with the data where GD read and re-read the transcripts taking note of any observations. Phase two involved a thorough, systematic, and complete coding of the data. Codes developed were data-derived or semantic to provide a succinct summary of the participants experiences. During phase one and two, a second researcher was recruited to identify potential researcher bias and to achieve richer interpretations of the data (Braun & Clarke, 2020). Both researchers coded 10% of the data independently before meeting to discuss areas of agreement and/or disagreement. Following this, GD analysed all transcripts independently. The process of theme development then occurred during phase three where GD examined the codes to identify similarity and overlap between patterns of semantic meaning and underlying concepts. Phase four involved reviewing the developed themes against the coded data to check whether the themes told a coherent story about the coded data. During this phase, met with the core research team to review the themes against the coded data. Once the themes were finalised, the fifth and sixth phase involved writing up the themes where GD created descriptions and a concise name to capture the story of the theme and how it relates to other themes and the overall research question. The theme descriptions and identified quotes were then reviewed by the core research team.

## Results

**Fig. 1:**
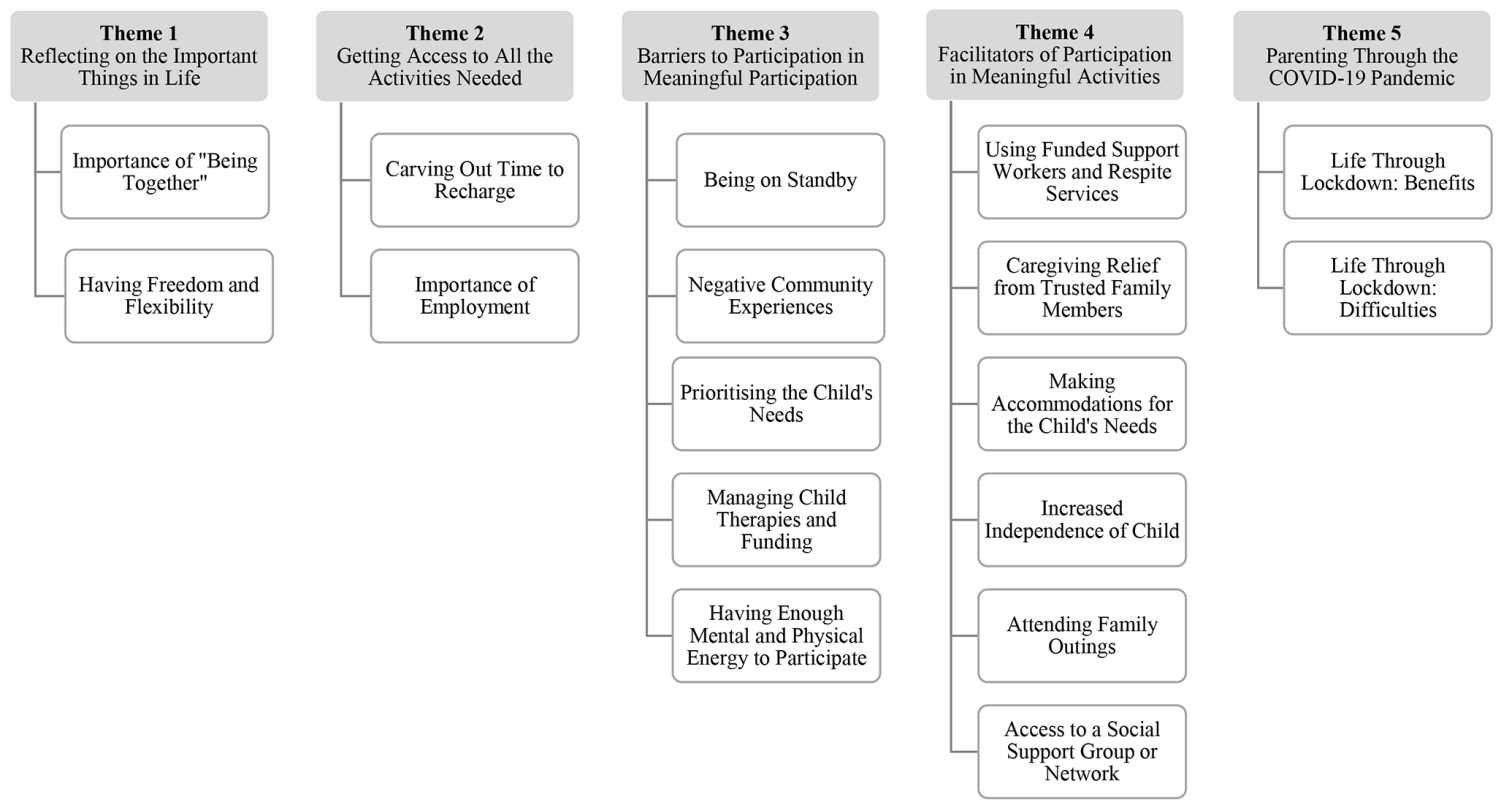
Thematic Map of Themes and Subthemes

Five themes with underlying subthemes were identified: (1) Reflecting on the important things in life; (2) Getting access to all the activities needed; (3) Barriers to participation in meaningful activities; (4) Facilitators of participation in meaningful activities; and (5) Parenting through the COVID-19 pandemic. Figure 1 outlines the structure of the themes and subthemes.

### 1. Reflecting on the Important Things in Life.

Parents identified a variety of factors and values they felt contributed to a good and well lived life. While some caregivers identified positive impacts on their life currently (e.g., a happy family), others highlighted the outcomes they hoped to have in future to achieve their perceptions of a good life (e.g., improved physical health).

#### Importance of “Being Together”

Most caregivers discussed strong family values, with their own happiness reported to be centred around their child/ren and family. Parenting their children brought lots of joy with caregivers identifying their relationship with their children and their autistic child’s development as the most important part of their life. *“To me, it’s all about family. As long as we’re together and we’re happy and doing things together, that’s all that matters”* (P08). Caregivers cherished the quality time they spent with their autistic child and family, identifying those moments as contributing positively to their life. *“When I see that happiness, when I see moments with Sienna and my husband and the dog, and we’re all just giggling together in the lounge room I think that’s what a happy and well lived life is for me”* (P09).

#### Having Freedom and Flexibility

Having freedom and flexibility was identified as an important indicator of a good and well lived life. This involved having autonomy and balance in daily activities as well as the freedom to access activities related to their own interests and/or self-care needs. *“I’d say one that has some meaningful relationships, freedom, and choice and control over what one wants to do.”* (P06). While a small group of the parents felt they had enough flexibility to meet their participation needs, most desired a better balance between caregiving responsibilities and freedom to engage in activities of interest to them. Parents felt that an increase in freedom would impact positively on their life through helping them to achieve personal goals, maintain good mental and physical health, and meet their social and recreational needs. *“My life will be a good one if I could actually do everything, well maybe not everything, most of the things that I want to do.”* (P02).

### 2. Getting Access to All the Activities Needed.

Participation in activities across leisure, social, and community contexts were identified as an important indicator of QoL. The positive impact of social relationships, leisure pursuits, and employment on their general wellbeing were highlighted, with those parents who were able to regularly access a range of activities feeling content with their life.

#### Carving out Time to Recharge

Caregivers highlighted the crucial need for *“carving out time to recharge”* (P06) and this was particularly important to caregivers of multiple children and/or children with higher support needs. Engaging in activities related to their own interests was suggested to be a form of self-care and was highlighted as having a positive impact on their mental and physical health and parenting. *“When I have access to just everything that is important to me, even if it’s only a little bit more than what I normally would have, it lifts me significantly and it also increased my ability to parent him in a really positive way.“* (P07).

Participation in leisure and social activities was only beneficial for parents and their QoL when they had access to what *they* identified as meaningful. For a lot of parents, this meant weighing up the cost and benefit of different activities and avoiding events that might add to their already high levels of fatigue and/or stress. *“It’s picking and choosing the right activities that will help to replenish for me personally.”* (P06).

Participation needs and interests were reported to change over the course of their parenting journey with some caregivers beginning to find time to focus on themselves and their own interests now that their children were past the early intervention stage and settled into school. *“Now I have to re-identify who I was as a person to be able to understand what my needs are now and what I want now compared to what I did back then.”* (P09).

#### Importance of Employment

Most caregivers identified employment as the most meaningful activity, with many highlighting the social benefits of the opportunity to engage with other adults. This was particularly highlighted by single parents. *“To have a part of my life where I could interact with peers… I love being around people.”* (P16). Although adding to the overall stress and time pressure, re-engaging in the workforce was a positive experience for caregivers with many identifying the fulfillment they received from their roles. *“I really like my job and that’s I guess a big part of my life is I feel like I make a real difference in people’s lives and so that’s quite fulfilling for me.”* (P11).

Caregivers who had already re-engaged in part- or full-time employment reported the benefits associated with work such as increased independence and the opportunity to contribute to society outside of their caregiving role. *“My quality of life wasn’t any worse and I think it got a lot better when I went to work so once my life didn’t just totally revolve around my son then I found myself a bit again.”* (P11). Many parents planning to re-engage with the workforce expressed the possibility of changing their career paths with some identifying new interests following their parenting experiences. *“Eventually I will work in a school and use the skills that I’ve got from my boys to be able to help other kids.”* (P14).

### 3. Barriers to Participation in Meaningful Activities.

Caregivers identified several barriers to accessing meaningful independent and family activities. Finding a balance became difficult as they managed caregiving, added responsibilities of child therapies and services, and their own physical and mental health.

#### Being on Standby

Being a primary caregiver often involved being the first point of contact for anything child related such as difficulties at school or liaising with therapists and support workers. This required parents to be available in case something unexpected happened when the child was in someone else’s care, making it difficult to commit to regular activities such as employment. *“I’ve never been able to go back to work since like Stefan was born because one thing or another came up that all my attention and focus needed to be on him and that even when I was dropping him at school there were times when I’d have to pick him up in the middle of the day.”* (P07). Parents also reported feeling restricted from making plans throughout the school day in case they were required to attend to their child. *“I can’t commit myself to anything because I don’t know whether or not Nathan’s gonna have a bad day and I’m going to be required to pick him up ten minutes after the bell is gone or half an hour before it’s gone off for the afternoon.”* (P19).

#### Negative Community Experiences

Accessing community activities was identified as challenging for a lot of parents. For some families, the child’s sensory difficulties and tolerance to environments outside of home (e.g., supermarket) made them avoid environments or activities as a family unit. *“We can’t go to big crowd, crowded areas because he can’t handle it, that kind of thing. We haven’t really done too much in terms of holidays as well because of that.”* (P10). Negative reactions or comments from strangers when out in public caused additional stress and anxiety for parents, limiting their community activities with their child. *“Even taking him to the supermarket, you’re already stressed before you went because are we going to have a meltdown and is the person behind you or somebody going to comment.”* (P08). For some parents, such experiences also extended to family gatherings, which were either perceived as stressful or something that parents could not attend, impacting on their ability to meet their participation needs. *“The main gap in my participation is social family events… we’ve ended up just giving up on like Christmas day.”* (P16).

#### Prioritising the Child’s Needs

Caregivers rarely put their own needs above those of their children with many identifying this as one of the factors reducing their ability to meet their participation needs. *“It’s that sort of sacrifice that happens I think when you have kids, you know that it’s not, it’s not your life anymore it really belongs to other little people.”* (P17). For some parents, their level of responsibility was high due to not having another parent or family member to rely on for respite or caregiving help. *“I even feel if, well the right person, shared the load then I would have more me time.”* (P01). For others, the effort needed to coordinate someone else to care for their child was enough to prevent them from regularly participating in activities solely for their own benefit. *“I think having three children obviously, as I say you don’t have enough hours in the day to, my kids take priority and it’s, it’s obviously, it is, it’s hard. It’s hard to plan activities that are just for your benefit alone.”* (P18).

#### Managing Child Therapies and Funding

Due to the National Disability Insurance Scheme (NDIS) being participant led, the administration responsibilities and funding management associated with child therapies and services meant caregivers identified this as a part- or full-time workload. Being a *“therapy manager”* (P12) increased the time pressure felt by parents, reducing their time available for self-care or leisure activities. *“Therapy is a full-time job whether you like it or not, that’s just how it is unfortunately yeah, and he needs, he needs the support so that’s, that’s my job for now.”* (P19). Most parents highlighted difficulty in navigating different services and working out how to use their NDIS funding. For some, not having the knowledge or time to research supports and services restricted their access to additional funding that could provide them with respite. *“They’ve given me funding for him to have a social worker to come to the house and spend time with him but I wouldn’t even know where to start.”* (P17).

Continuity of services was also identified as an issue, with the time needed to research and find new services and then waiting for that service to become available, further increasing the time pressure experienced by parents. *“Navigating …Hayden’s program, the NDIS supports and navigating what, you know, the best practice in treatment is and balancing that with my need for respite, it’s a really tricky one.”* (P16). This process was particularly difficult for families with more than one autistic child engaging in therapy, often requiring the primary caregiver to thoroughly plan out the week. *“I spend most of my time running children…just juggling that alone has been pretty hectic but we probably do two therapy sessions, no three therapy sessions a week across both boys.”* (P14).

#### Having Enough Mental and Physical Energy to Participate

Some caregivers felt they were unable to engage in activities related to their own interests due to feeling fatigued or experiencing physical or mental health problems. *“Between working and helping my husband in his business and having four children, let alone all their extra needs and therapy and everything else, they just, you just never felt like you have enough energy.”* (P04). Instead, caregivers would prioritise rest or self-care related activities when their child attended school or was in someone else’s care. *“I find maintaining relationships since having Josh extremely exhausting. When I get a text saying what are you doing on Wednesday, I get really anxious. I’m like oh my god, I don’t want to do anything.”* (P01). Further, increased fatigue, stress, and physical injuries resulting from caregiving restricted some parents from re-engaging in employment. *“I was exhausted and I kept injuring my back so work became something that I could never even contemplate going back to.”* (P07).

### 4. Facilitators of Participation in Meaningful Activities.

A number of factors were identified by caregivers that allowed them to engage in their own interests and activities, with some attributing this to their positive outlook on life.

#### Using Funded Support Workers and Respite Services

Parents highlighted the beneficial impacts of employing support workers or using respite programs for their child via NDIS funding. Being able to leave their child in someone else’s care provided parents with a break from caregiving to engage in activities meaningful to them. *“I’ve got those support workers and I had my first night away from Brad for a work function.”* (P05). Further benefits were reported by parents who had support workers accompany them on family outings. Having additional caregiving help when out in the community provided parents with increased confidence in attending outings or running errands with their children and making sure their child was safe. *“Even though he’s seven I can’t turn a blind eye on him. I have 100% be on the ball so if my husband’s not with me, I always make sure that I’ve got the support worker with me.”* (P03).

#### Caregiving Relief from Trusted Family Members

Parents highlighted the importance of having a spouse or other family members to rely on for caregiving relief as this provided them with a break and time to participate in activities meaningful to them. *“They’ll pick him up, mum cooks for him, she’ll cook for us so that’s all helpful… that’s where my pressure is a bit eased in that regard.”* (P13). This was particularly beneficial if the support was from someone who was familiar with the child and their caregiving needs, reducing the worry associated with leaving the child in someone else’s care. *“I can leave them with her and she knows Oscar like I know Oscar so I don’t have to explain things to her, I don’t have to get her to understand what’s happening.”* (P17). Being able to leave the child with a trusted family member during the day or overnight provided parents with increased opportunity for participation in activities related to employment, leisure, and social contexts. *“I will drop them at school every day and then Nanny and Pop do the other bits of picking up… the school holidays they definitely help because we both work full time.”* (P11).

#### Making Accommodations Based on the Child’s Needs

Parents identified the importance of having a deep understanding of their child’s sensory needs as this allowed them to continue accessing the community with their child and wider family unit. *“One of the reasons why we have been so successful together as a family is that we were good at remoulding ourselves constantly and to, from the beginning to whatever Sienna needs.”* (P09). Planning was a crucial step for a lot of parents and something that had *“become second nature”* (P12). For some, this often involved adapting activities to cater for their child’s needs such as visiting public areas at quiet times. *“Instead of going to a restaurant at peak time, we would go a bit later when the restaurant was a bit quieter and we would sit in the corner area, we would turn him and sit him facing the wall so he wouldn’t be too overstimulated with the crowds around us.”* (P03). Other families relied on different tools (e.g., noise cancelling headphones, iPads) to help manage their child’s needs or behaviour when out in public.

#### Increasing Independence of Child

Some parents highlighted the increased flexibility with their child’s increasing independence and ability to manage their own sensory needs. Such improvements reduced the vigilance needed, providing caregivers with extra time for their own interests and activities. Some contrasted this with the difficulties experienced during the early years following diagnosis and navigating therapies, understanding their child’s needs, and learning about autism. *“When he was younger we, I guess we picked and chose and we adapted to his needs then, whereas today we don’t have to so we participate and do everything that we would like to do as a family.”* (P08). Further benefits were identified by parents whose children had begun to manage their own sensory needs with parents reporting increased ease in accessing different community or leisure related activities with their child. *“He’s learnt to manage whatever sensory concerns he has, he’s learnt to manage school and on the off occasion we have to take him out.”* (P14).

#### Attending Family Outings

Some caregivers felt they satisfied their participation needs through fitting in activities alongside their caregiver responsibilities. This often meant planning social outings with other families so they could supervise their child while also interacting with their friends. *“We were going to a play centre so I’d let the kids go nuts in the play centre and we’d have a quick coffee”* (P20). This approach was particularly helpful for parents without caregiving support from other family members or funded services. *“When I go to the park I invite my girlfriends that have kids so that when they’re playing, if we’re sitting down there’s a bit of mum time there.”* (P03).

Other parents reported the positive impact of spending quality time with their family at home or out in the community, with some identifying those family activities as their most meaningful participation. *“Weekend’s family time, that’s what we spend together… whether it’s just going into the city to do something or going for a bushwalk or heading down the coast to do something, we do something together.”* (P08).

#### Accessing a Social Support Group or Network

Caregivers identified the positive impact of engaging with community support groups, particularly if they were a single parent. For most parents, this involved attending a social support group for parents of autistic children, where they could meet others who understood their experiences, encouraging and/or helping them to get back out in the community. *“Connecting with people who were in a similar space sort of gave me the strength to then go, we can have a life that’s outside of you know, outside of caring and parenting.”* (P07).

### 5. Parenting Through the COVID-19 Pandemic.

The pandemic and subsequent lockdowns had a mixed impact on families. Although stay-at-home orders did not impact participation habits for some families due to already low participation levels, the majority reported the strain of increased time spent caring for their child and family as the lockdown period extended.

#### Life Through Lockdown: Benefits

For some families, lockdown was welcomed as it provided parents with a break from the time commitments associated with their child’s services and therapies allowing parents’ increased time to rest and to engage in more at home leisure. *“I actually really enjoyed at least the first part of the lockdown last year in March till I think June because that, that’s it we finally got the downtime we needed, or I needed.”* (P02). Further benefits were identified by caregivers who felt the work-from-home and home-schooling restrictions forced their family to spend more quality time together. *“There’s been a lot of positive things, we’ve got to spend time as a family and we eat dinner all together, that’s always nice.”* (P14).

#### Life Through Lockdown: Difficulties

For many parents, the extended lockdown periods were reported to have a negative impact on their mental and physical health. Parents who regularly attended their own leisure and social activities reported the increased pressure and stress due to being unable to get a break from caregiving responsibilities. *“It has certainly impacted me as well from a mental health perspective. Even though what I do access is limited, it’s still like super important in my life.”* (P07).

Further difficulties were faced by parents who were required to support their child’s remote learning, ultimately reducing the time they had for their own self-care or interests. *“I would use the time that the boys are at school to do things for myself so that [home-schooling] has really impacted on my, the time that I get.”* (P15). This was particularly difficult for parents with more than one child or those who needed to fit the school related tasks alongside their own employment responsibilities. *“I can only do so much with him at home because I’m working from home, I have my older son that I’m home-schooling as well.”* (P17).

## Discussion

The overall study aim was to examine primary caregiver participation in leisure, employment, and community related activities when raising autistic children, and their associated QoL. The findings highlight that although meaningful participation is important for QoL the ability of primary caregivers of autistic children to access a desirable range of activities is often limited as they devote their time and energy into supporting their child. While prior research often associates parenting autistic children with negative caregiver outcomes (Frazier et al., 2020; Vasilopoulou & Nisbet, 2016), caregivers in the current study identified their parenting role and their child as contributing positively towards their life and an important aspect of their subjective QoL. Parents in the current study largely attributed low participation rates and QoL outcomes to time pressure and the lack of support for caregiving. Thus, it was not their autistic child that placed burden on them, but rather *the lack of support and resources parents needed* to help them and their child thrive and access a community that is not designed to meet and accept their child’s differing needs.

### Participation and Quality of Life

Achieving balance in everyday life and activities was reported by caregivers to be important for their subjective QoL. Parents highlighted the need for flexibility and freedom to access desired activities with having free time for themselves outside of caregiving identified as crucial to their health and well-being. This is consistent with prior research that suggests the importance of participation in a variety of everyday activities (Law, 2002) with occupational balance demonstrated as having a positive impact on health, well-being, and life satisfaction in the general population (Håkansson et al., 2006; Stein et al., 2011; Wagman et al., 2012). Research examining lifestyle balance (i.e., time spent between work and home) also suggests the importance of being able to sufficiently manage multiple demands of time to achieve goals and create opportunity for personal renewal (Matuska & Christiansen, 2008). Given the limited time available for leisure and self-care activities reported by parents in the current study, it is not surprising that most also parents felt they had reduced QoL. These findings highlight the need for more balance in daily activities for caregivers raising autistic children as increasing opportunities for participation in activities relating to their own interests and self-care may help to improve their QoL outcomes.

Participation in meaningful activities was reported to be more important for caregiver QoL than the frequency of participation. Caregivers highlighted the need to manage their already high levels of fatigue through choosing to participate in activities that would help them to recharge or add to value to their QoL. Several meaningful activities were identified by parents with the most common being employment, family outings, and leisure activities. Past literature has also highlighted similar activities as being beneficial for QoL in caregivers of children with additional needs, including autism (Davy et al., 2022), with increased frequency of health-related activities associated with higher levels of maternal QoL (Bourke-Taylor et al., 2012; Harris et al., 2022). However, other research has found that increased participation in leisure and community activities resulted in poorer physical health-related QoL in mothers of autistic children (Rizk et al., 2011). Such findings suggest the importance of helping caregivers to increase opportunities for activities related to health and self-care without adding to their already busy schedules.

### Meeting Occupational Needs

Being the primary caregiver often required a delicate balance between meeting the caregiving needs of their child, supporting their family, and managing their own mental and physical energy. Prior literature also highlights the challenges that primary caregivers face due to the increased time pressure experienced, with mothers of autistic children seen to spend significantly more hours in caregiving tasks compared to mothers of non-autistic children (Smith et al., 2010). Further, despite at least half of the sample in the current study living with a partner and being employed, all primary caregivers reported taking on the majority of the caregiving responsibilities including management of child funding, therapies, and any school related issues. Increased caregiving time by fathers has been associated with improved maternal QoL in families of autistic children (Ozgur et al., 2018). Therefore, improving the balance of parenting responsibilities may not only help to alleviate some of the time pressure experienced by primary caregivers but also improve their access to desired occupations (e.g., employment, leisure) and QoL.

Prior research has highlighted access to respite as a crucial support for parents of children with additional needs as it provides parents with temporary relief from caregiving related tasks, allowing them time for rest or other desired activities (Dyches et al., 2016; Strunk, 2010). However, accessing quality respite services has been shown to be an issue for families of autistic children, particularly those in low income or rural areas (Cooke et al., 2020). Difficulties accessing respite and/or support workers was highlighted by caregivers in the current study due to the lack of respite services available and the time needed to source providers and/or employ workers through their government funding. Continuity of services was also identified as an issue by some parents due to constant changes in therapists and support workers requiring parents to continually source new service providers. Thus, although families of autistic children were provided with funding for child related services and supports, there are barriers associated with accessing quality respite and support services. Therefore, improving the resources available to assist parents of autistic children utilise their child’s funding may help to further alleviate the time pressure associated with caregiving, particularly for families who don’t have childcare or social support from extended family members or friends.

The COVID-19 pandemic and stay-at-home orders had mixed impacts on families with many parents reporting the initial lockdown period as positive due to the increased time for at home leisure and/or a break from child related services and therapies. However, as the lockdown period extended, parents highlighted the strain of balancing home-schooling, care for their child/ren, and work-from-home arrangements. Similar findings have been reported in other studies suggesting that families of autistic children experienced increased negative QoL and stress outcomes compared to families of non-autistic children throughout the COVID-19 pandemic (Kalb et al., 2021; Pellicano et al., 2022; Ueda et al., 2021).

### Potential Implications

The findings from the current study highlight the role that participation may have on primary caregiver QoL in families of autistic children. When working with families, clinicians should consider the parent and wider family unit when supporting or providing services for the child. Moreover, the likely impact on occupational balance should be raised with families to help them adjust, plan, and prepare the necessary supports. Further, more supports are needed to help primary caregivers to create time alongside their caregiving responsibilities for occupations (e.g., leisure, employment, social activities) deemed meaningful for their own QoL. A recent study demonstrated the benefits of focusing a portion of the child’s occupational therapy session to coaching mothers of children with additional needs, including autism, to engage in leisure and recreational activities with improvements found in caregiver participation and QoL outcomes after six sessions (Harris et al., 2022). Thus, integrating supports for the caregiver into their child’s services may help to improve caregiver outcomes without needing to add another task to their already busy schedules.

Additional supports also need to be developed to help parents manage child related services and therapies. While funding has been provided to help families of autistic children improve outcomes for their child, more support is needed to educate parents on how to manage their support funding and seek appropriate services or supports. Further, resources on the different services available to help caregivers of autistic children would be beneficial, particularly post diagnosis when most parents are first learning about autism and what it may mean for their child and family.

## Limitations and Future Directions

A possible limitation in the current research was that many of the caregivers interviewed were of autistic children diagnosed early at 2.5 years of age or younger. As previous research suggests the beneficial impact of early diagnosis on child school aged outcomes (Clark et al., 2018), the parenting experience of caregivers in the current study may be different to families of autistic children who were diagnosed later. In addition to this, the majority of the children in the current study were male, which may limit our understanding of any unique factors that caregivers of female autistic children experience in raising their child. Therefore, future research may benefit from examining the influence of different child-related factors, including gender, on caregiver outcomes.

All caregivers interviewed in the current study were primary caregiving mothers. Although prior research demonstrates reduced QoL in mothers of autistic children compared to fathers (McStay et al., 2014b), the role of participation on QoL in fathers and/or secondary caregivers remains relatively unknown (Davy et al., 2022). Therefore, further investigation into the occupational experiences of both primary and secondary caregivers of autistic children would help to improve our understanding of how best to support both parents of autistic children in achieving good QoL.

Due to the nature of the interview, parents may have felt reluctant to be completely honest about their perspectives potentially leading to biased responses. To mitigate this likelihood, a strong attempt was made by the interviewer (GD) to build rapport with parents’ during their involvement in the main longitudinal study from which the sample was drawn. Moreover, parents were encouraged to speak openly throughout the interview and ensured that all identifying information was kept confidential.

Finally, data collection for the current study was carried out during the COVID-19 pandemic (in 2021) with some interviews conducted during the stay-at-home orders. Given the increased negative impact of COVID-19 on participation and QoL, particularly for families of autistic children (Pecor et al., 2021), the experiences, perceptions, and beliefs relating to caregiver participation and QoL are expected to have been influenced, and may not be completely generalizable to non-pandemic times.

## Conclusion

Overall, the current study demonstrates the important role that leisure, community, and workforce participation can have on perceived QoL in primary caregivers of autistic children. While caregivers recognise the beneficial impact of participation in meaningful activities for their own QoL, their ability to access a desirable range of activities is often limited as they devote their time and energy into bettering their child’s life. These findings suggest that families of autistic children need support in managing the increased time pressure associated with caregiving. Supporting parents, particularly primary caregivers, to create time for themselves to re-engage in activities that are meaningful to them may help to improve their QoL and associated family outcomes.
